# Differences in tumor mutational burden assessment: a national ring trial

**DOI:** 10.2340/1651-226X.2026.45220

**Published:** 2026-03-04

**Authors:** Christine Federspiel Secher, Christina Westmose Yde, Britt Elmedal Laursen, Estrid Høgdall, Henriette Sylvain Thomsen, Tim Svenstrup Poulsen, Mads Sønderkær, Tina Kringelbach, Mads Malik Aagaard Jørgensen, Rikke Frydendahl Sick Olsen, Anja Elaine Sørensen, Christian Baudet, Magnús Halldór Gíslason, Michael Knudsen, Ida Kappel Buhl

**Affiliations:** a Department of Oncology, Copenhagen University Hospital, Rigshospitalet, Copenhagen, Denmark; b Department of Genomic Medicine, Copenhagen University Hospital, Rigshospitalet, Copenhagen, Denmark; c Department of Molecular Medicine, Aarhus University Hospital, Aarhus, Denmark; d Department of Oncology, Aarhus University Hospital, Aarhus, Denmark; e Molecular Unit, Department of Pathology, Herlev & Gentofte University Hospital, Herlev, Denmark; f Institute of Clinical Medicine, Faculty of Health and Medical Sciences, University of Copenhagen, Copenhagen, Denmark; g Department of Molecular Diagnostics, Aalborg University Hospital, Aalborg, Denmark; h Department of Clinical Genetics, Vejle Hospital, Vejle, Denmark; i Department of Pathology, Odense University Hospital, Odense, Denmark; j Department of Pathology, Zealand University Hospital, Roskilde, Denmark

**Keywords:** Tumor mutational burden, immune checkpoint inhibitors, high-throughput nucleotide sequencing, whole genome sequencing, exome sequencing

## Introduction

Tumor mutational burden (TMB) quantifies the number of somatic mutations per megabase (mut/Mb) in a tumor genome and is used clinically as a predictive biomarker for response to immune checkpoint inhibitors (ICIs) across multiple cancer types [1–3]. The U.S. Food and Drug Administration (FDA) has approved TMB as a tissue-agnostic indication for treatment with the PD-1 inhibitor pembrolizumab of unresectable or metastatic TMB-high solid tumors (≥10 mut/Mb), as measured by an FDA-approved test [4].

A 2023 survey within the Danish Comprehensive Cancer Center (DCCC) Variant Interpretation Network revealed substantial variation in TMB calculation across centers, including differences in next-generation sequencing (NGS) methods, such as whole-genome sequencing (WGS), whole-exome sequencing (WES), and targeted panels, as well as variation in bioinformatic filtering.

TMB is used as one of several biomarkers for inclusion in ProTarget, a Danish national investigator-initiated phase 2 trial [5]. In ProTarget, TMB estimates were accepted based on local testing, raising questions about cross-laboratory alignment. This led to the initiation of the Danish National TMB Ring Trial, designed to evaluate variation in TMB scoring across sequencing platforms and bioinformatic methods using an international reference standard.

## Methods

This was an unblinded ring trial conducted through the national DCCC Variant Interpretation Network to assess TMB calculation across laboratories in Denmark. All seven laboratories at major public hospitals that routinely report TMB were invited to participate.

Reference samples from SeraCare (Seraseq® genomic DNA (gDNA) or formalin-fixed paraffin-embedded (FFPE) TMB Mix Score 7, 9, 13, 20, and 26) were sequenced using locally applied NGS platforms for *in silico* prediction of TMB values.

Sequencing approaches included WES with two different capture kits (TWIST or Agilent SureSelect Clinical Research V2), WGS (Illumina PCR-free WGS), and targeted sequencing using the Ion Torrent Oncomine Comprehensive Assay Plus (OCA+) or the Illumina TruSight Oncology 500 (TSO500) panels. Sequencing data were shared with all participating sites via the National Genome Center High-Performance Computer.

Participating centers were required to analyze and report results for SeraCare TMB scores 7, 9, and 13, while 20 and 26 were optional. Sites were encouraged to test and submit results from multiple bioinformatic pipelines and to explore different filtering strategies. All participating sites were also asked to complete a standardized spreadsheet documenting methodological and bioinformatic details for each submitted TMB value and to note where in-house routine pipelines were used.

## Results

The ring trial was conducted in 2024 and included all seven invited sites. In total, 24 result sets were submitted, comprising data from WGS, WES, and targeted panel sequencing TSO500 and OCA+. A result set consisted of SeraCare TMB scores 7, 9, and 13 (with 20 and 26 where available), measured using the same method. Three submitted result sets from OCA+ were generated from FFPE samples, whereas the remaining analyses were performed on gDNA.

**Figure 1:**
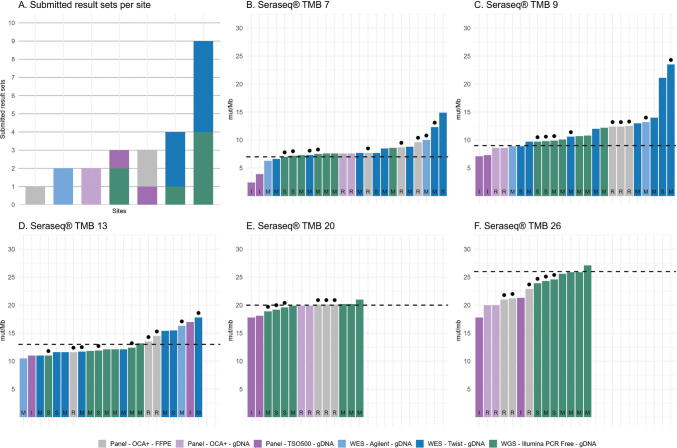
(A) Distribution of submitted result sets per site, categorized according to sequencing method and tissue type. (B–F) TMB results for SeraCare reference standards 7, 9, 13, 20, and 26. Samples analyzed using in-house routine pipelines are marked with a filled black circle above the bar. Targeted panels were run on tumor-only samples. WES and WGS were performed on matched tumor–normal pairs. Variant caller: I = Illumina, R = Ion Reporter, M = Mutect2, S = Strelka2. Abbreviations: Agilent, Agilent SureSelect Clinical Research Exome V2; FFPE, formalin-fixed paraffin-embedded; gDNA, genomic DNA; mut/Mb, mutations per megabase; OCA+, Ion Torrent Oncomine Comprehensive Assay Plus; PCR, polymerase chain reaction; TMB, tumor mutational burden; TSO500, Illumina TruSight Oncology 500; Twist, Twist Human Comprehensive Exome Panel; WES, whole-exome sequencing; WGS, whole-genome sequencing.

Figure 1A illustrates the distribution of submitted result sets across sites, categorized according to sequencing method and tissue type. Figure 1B–F illustrates the broad range of outcomes across the SeraCare reference standards with expected TMB values of 7, 9, 13, 20, and 26 mut/Mb. The results revealed substantial variability in measured TMB values, even when restricting the comparison to in-house routine pipelines (bars marked with a filled black circle above). Choice of variant caller differed across participating sites (Mutect2 [6], Strelka2 [7], Illumina TSO500 v.1.1, Illumina TSO500 v.2.2 Local App and Ion Reporter version 5.18 and 5.20), as indicated by letters within the bars. The accompanying spreadsheet revealed additional methodological differences not shown in the figure. For WES and WGS analyses, both tumor and matched normal samples were available, whereas targeted panel analyses were performed as tumor-only. Further differences included the inclusion or exclusion of synonymous variants, variation in the size and content of the analyzed genomic regions, the use of normal filtering, variant allele frequency cut-offs, and the number of supporting reads. Overall, TMB estimates were more consistent and accurate for samples with higher expected TMB values, whereas greater variability was observed among samples with lower TMB. Across sequencing platforms, duplicate and triplicate analyses yielded highly similar TMB values, indicating high technical reproducibility within individual workflows (data not shown).

## Discussion and conclusion

The Danish National TMB Ring Trial demonstrated substantial variability in TMB assessment due to methodological differences and highlighted the diversity of approaches used for TMB calculation. Variability was most pronounced at TMB values between 7 and 13 mut/Mb, which can have direct clinical consequences, since a high TMB value (often defined as ≥10 mut/Mb) often qualifies a patient for treatment with ICIs [5]. Following the results of the ring trial, one site updated its routine in-house pipeline to exclude synonymous variants in order to achieve TMB scores that were more consistent with those from other sites.

Beyond highlighting analytical discrepancies, the trial improved data quality across participating sites and strengthened national collaboration within the DCCC Variant Interpretation Network. Through shared analyses and transparent discussion of results, the network established a foundation for data sharing and initiated efforts to harmonize TMB measurement. Importantly, the TMB ring trial was not intended as a validation of individual laboratory assays, nor was it powered to draw general conclusions about optimal analytical approaches. Rather, it was a comparative analysis across laboratories aimed at highlighting the challenges of applying a biomarker whose measurement lacks standardization. The study represents a critical step toward identifying inconsistencies and promoting best practices in assay setup, bioinformatic processing, and result interpretation. A robust, technology-independent evaluation of TMB is essential to support clinical decision-making, ensure consistent inclusion in clinical studies, and enable meaningful comparison of results at both national and international levels [8, 9]. Here, we suggest the use of an international reference standard to assess comparability.

In conclusion, the Danish National TMB Ring Trial successfully established a sustainable platform for knowledge sharing and collaboration within the national DCCC framework and provides a foundation for future efforts to validate, standardize, and harmonize TMB assessment.

## Data Availability

Data presented in the paper are available upon reasonable request.
